# Functional Genomics Unique to Week 20 Post Wounding in the Deep Cone/Fat Dome of the Duroc/Yorkshire Porcine Model of Fibroproliferative Scarring

**DOI:** 10.1371/journal.pone.0019024

**Published:** 2011-04-20

**Authors:** Loren H. Engrav, Christopher K. Tuggle, Kathleen F. Kerr, Kathy Q. Zhu, Surawej Numhom, Oliver P. Couture, Richard P. Beyer, Anne M. Hocking, Gretchen J. Carrougher, Maria Luiza C. Ramos, Matthew B. Klein, Nicole S. Gibran

**Affiliations:** 1 Department of Surgery, Division of Plastic Surgery, University of Washington, Seattle, Washington, United States of America; 2 Department of Animal Science, Iowa State University, Ames, Iowa, United States of America; 3 Department of Biostatistics, University of Washington, Seattle, Washington, United States of America; 4 Department of Surgery, Ramathibodi Hospital, Mahidol University, Bangkok, Thailand; 5 Department of Environmental and Occupational Health Sciences, University of Washington, Seattle, Washington, United States of America; 6 Department of Surgery, Division of Plastic Surgery, Federal University of São Paulo, São Paulo, Brazil; 7 Department of Surgery, University of Washington, Seattle, Washington, United States of America; Health Canada, Canada

## Abstract

**Background:**

Hypertrophic scar was first described over 100 years ago; PubMed has more than 1,000 references on the topic. Nevertheless prevention and treatment remains poor, because 1) there has been no validated animal model; 2) human scar tissue, which is impossible to obtain in a controlled manner, has been the only source for study; 3) tissues typically have been homogenized, mixing cell populations; and 4) gene-by-gene studies are incomplete.

**Methodology/Principal Findings:**

We have assembled a system that overcomes these barriers and permits the study of genome-wide gene expression in microanatomical locations, in shallow and deep partial-thickness wounds, and pigmented and non-pigmented skin, using the Duroc(pigmented fibroproliferative)/Yorkshire(non-pigmented non-fibroproliferative) porcine model. We used this system to obtain the differential transcriptome at 1, 2, 3, 12 and 20 weeks post wounding. It is not clear when fibroproliferation begins, but it is fully developed in humans and the Duroc breed at 20 weeks. Therefore we obtained the derivative functional genomics unique to 20 weeks post wounding. We also obtained long-term, forty-six week follow-up with the model.

**Conclusions/Significance:**

1) The scars are still thick at forty-six weeks post wounding further validating the model. 2) The differential transcriptome provides new insights into the fibroproliferative process as several genes thought fundamental to fibroproliferation are absent and others differentially expressed are newly implicated. 3) The findings in the derivative functional genomics support old concepts, which further validates the model, and suggests new avenues for reductionist exploration. In the future, these findings will be searched for directed networks likely involved in cutaneous fibroproliferation. These clues may lead to a better understanding of the systems biology of cutaneous fibroproliferation, and ultimately prevention and treatment of hypertrophic scarring.

## Introduction

After deep partial-thickness injury to the skin, it is common for patients to heal with fibroproliferative (hypertrophic) scarring. Although the condition has been recognized at least since 1893 [1] and studied in detail, there is still no effective prevention or treatment. The family of fibroproliferative disorders includes cirrhosis, idiopathic pulmonary fibrosis, myelofibrosis, atherosclerosis, rheumatoid arthritis, postsurgical adhesions, corneal fibrosis, Dupuytren's disease and the three dermal variants, fibroproliferative scarring, keloid formation and scleroderma. Tredget reviewed these dermal conditions in 1994 [2] and in 2009 [3], making it clear that in the 15 years between reviews no real advances in prevention or treatment occurred. Scarring is a topic of interest not only to burn researchers; Broerse has reported that burn survivors also rank it among the most undesirable outcomes to be solved [4].

Progress is hindered by lack of understanding of the transcriptomics, proteomics, and metabolomics (the systems biology) of cutaneous fibroproliferation, which prevents directed research towards prevention/treatment. There are several barriers.

1. There has been no accepted and validated *in vivo* animal model of fibroproliferation that permits detailed study of the effects of shallow and deep partial-thickness wounds in pigmented and non-pigmented skin, over time [5], [6], [7], [8], [9], [10], [11], [12], [13], [14], [15], [16], [17], [18], [19], [20]. Few animals produce fibroproliferative scar. Using shallow partial-thickness wounds as the control is important to eliminate the events of normotrophic healing and few models permit the use of simultaneous shallow and deep partial-thickness wounds. Comparing pigmented and non-pigmented skin is important, as the prevalence of fibroproliferative scarring is higher in pigmented skin; few models include pigmented and non-pigmented skin. Studying the process over time is important, as it is not known when the process begins and few models permit time studies.
2. The historical lack of an accepted animal model of cutaneous fibrosis means that human tissues have been the only source for study. However, it is impossible to obtain human tissue immediately and serially after injury or onset of disease in a given patient. The majority of studies have analyzed tissues obtained months or years after injury or onset, long after early signaling has ceased, and in the case of burns, with no control of depth of injury, i.e. superficial or deep partial-thickness. Robson [21] has demonstrated that time is an important variable in wound repair. Cole [22] reported that relevant gene expression might be early and transient. Early and serial study of the fibroproliferative process is crucial, yet is not possible with human tissue.
3. Studied human fibroproliferative tissue has been minced and homogenized. This destroys skin microanatomy and mixes all cell populations. Since superficial partial-thickness injury does not produce fibroproliferative scar, but deep partial-thickness injury does [23], [24], [25], [26], the two dermal levels and the microanatomical locations of skin must be kept intact and distinct, so that events unique to each may be recognized and interactions identified.
4. The final reason we have failed to understand cutaneous fibroproliferation is that the process is probably multifactorial; gene-by-gene study is likely incomplete. Northern blot analysis and in situ hybridization have been used to evaluate gene expression, but these methods are time consuming and detect only a few genes per assay. Thus, a systems biology, genome wide, approach is needed.

We have assembled a system that overcomes these barriers. It includes 1) a validated large animal, Duroc (pigmented, red, fibroproliferative)/Yorkshire (non-pigmented, white, non-fibroproliferative) porcine *in vivo* model of cutaneous fibroproliferation that enables the study of two significant variables simultaneously over time, wound depth and skin pigmentation, making it unique among fibrosis models [27], [28], [29], [30], [31], [32], [33], [34] (confirmed by Hart [35], [36], [37], [38], [39], [40], [41], [42], [43], [44], [45], [46]), 2) laser capture microdissection that enables the study of the microanatomical locations of skin, thus avoiding homogenization of the entire organ, and 3) annotation of the Affymetrix Porcine GeneChip® enabling global profiling.

**Figure 1:**
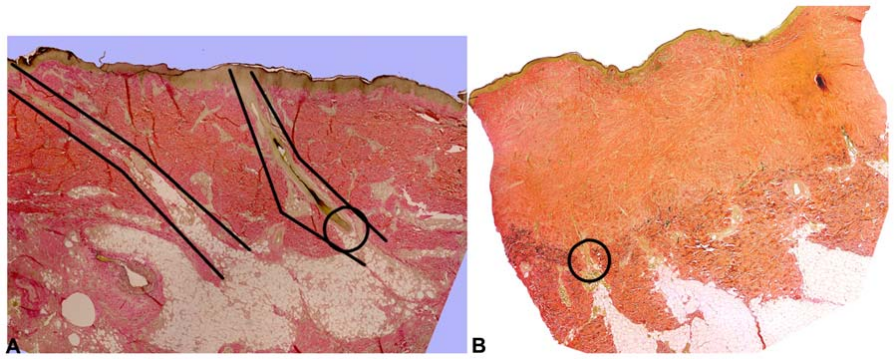
Cone/fat domes of the skin. Panel A is uninjured human skin. Two cones are schematically outlined and the deep cone/fat dome is circled. Panel B is human hypertrophic scar. The cone/fat dome deep to the scar is circled. (reprinted with permission of John Wiley & Sons including open access and a creative common license).

**Figure 2:**
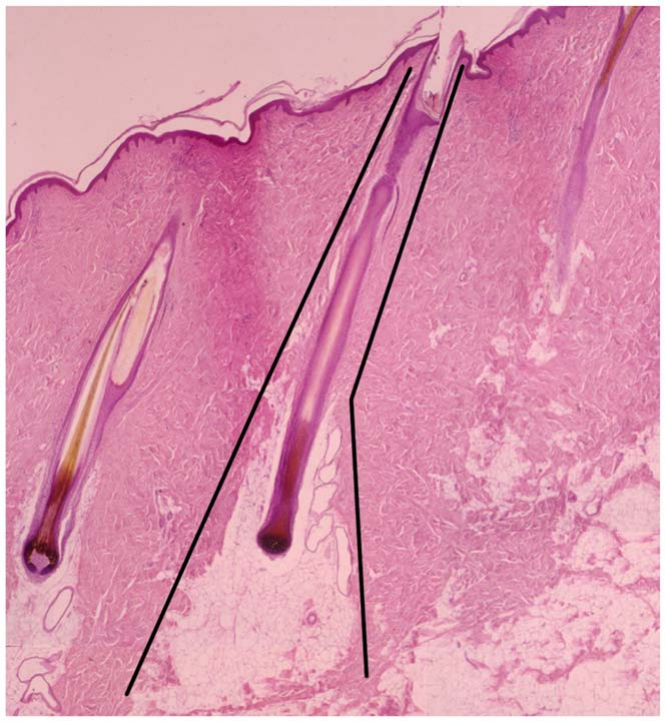
Cone/fat dome outlined in uninjured Duroc skin. The cone/fat dome structure is present in Duroc and Yorkshire skin (reprinted with permission of John Wiley & Sons including open access and a creative common license).

Using this system we have focused on one microanatomical location, the deep cone/fat dome. The cones of skin (Fig. 1a) were described by Shoemaker in 1905 [47] and again by Jackson in 1953 [48]. Matsumura [49], [50] and Zhu [27] revisited the cones. The deep cone structure including the fat dome is present on those body parts where hypertrophic scarring occurs, i.e. the cheek, neck, chest, abdomen, back, buttock, arm, forearm, dorsum hand, thigh, leg, dorsum foot, helical rim and ear lobe. Conversely, deep cones/fat domes are not present on those body parts where hypertrophic scar does not occur, including the scalp, forehead, concha, eyelid, palm and sole. The deep cones/fat domes are not present in the early human fetus nor are they present on the rat and rabbit and these do not form hypertrophic scar. On the other hand, pigskin does include deep cones/fat domes (Fig. 2). This structure is present beneath partial-thickness burns and human hypertrophic scar (Fig. 1b) and with the deep matrix, is all that remains of the dermis after deep partial-thickness injury; it warrants investigation as having a role in the fibroproliferative process.

We herein report the differential transcriptome over time and the functional genomics unique to Week 20 post wounding, from the deep cones/fat domes in the Duroc/Yorkshire porcine model of fibroproliferative scarring following shallow and deep partial-thickness wounds.

## Materials and Methods

Some aspects of this section were described and reported by Zhu [34]. If previously reported, we briefly summarize here.

### The Model

**Figure 3:**
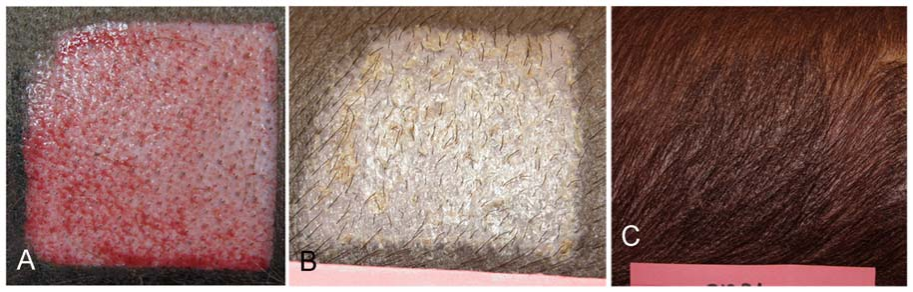
Shallow Duroc wound at time of wounding, 3 weeks and 20 weeks post wounding. Shallow Duroc wound at time of wounding (Panel A) and three weeks (Panel B) and twenty weeks (Panel C) post wounding. The wound is healed at 3 weeks and at 20 weeks there is no contraction or thickening and hair growth is full with minimal hyperpigmentation.

**Figure 4:**
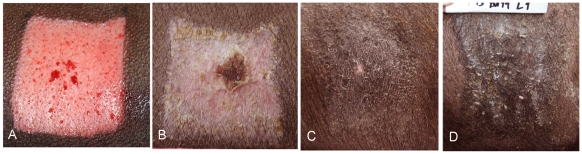
Deep Duroc wound at time of wounding and 3, 20 and 46 weeks post wounding. Deep Duroc wound at time of wounding (Panel A) and three (Panel B), twenty (Panel C) and forty-six weeks (Panel D) post wounding. The wound is not completely healed at 3 weeks and at 20 weeks there is contraction, thickening, no hair growth and hyperpigmentation. The contraction, thickening, absent hair and hyperpigmentation remain or have progressed at 46 weeks.

**Figure 5:**
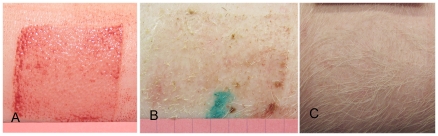
Shallow Yorkshire wound at time of wounding, 3 weeks and 20 weeks post wounding. Shallow Yorkshire wound at time of wounding (Panel A) and three (Panel B), and twenty weeks (Panel C) post wounding. The wound is healed at 3 weeks and at 20 weeks there is no contraction, thickening or hyperpigmentation and full hair growth.

**Figure 6:**
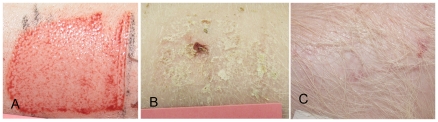
Deep Yorkshire wounds at time of wounding, 3 weeks and 20 weeks post wounding. Deep Yorkshire wound at time of wounding (Panel A) and three (Panel B), and twenty weeks (Panel C) post wounding. The wound is healed at 3 weeks and at 20 weeks there is no contraction, thickening or hyperpigmentation and full hair growth.

**Figure 7:**
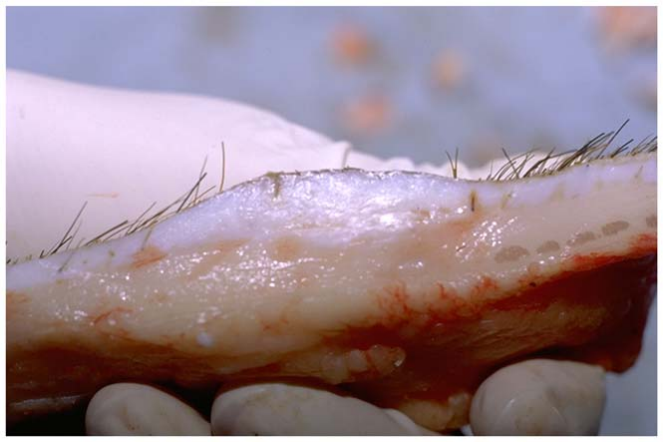
Duroc scar following a deep wound of 0.045″. The thick scar is visible at five months following a deep Duroc wound.

The experimental wounds were deep partial-thickness, leaving the deep portion of the dermal matrix and the deep aspect of the cones. The control wounds were shallow partial-thickness. The Duroc pigmented breed forms thick, fibroproliferative scar and was the experimental breed. The Yorkshire non-pigmented breed heals without fibroproliferative scar and was the control breed. Shallow and deep partial-thickness, Duroc and Yorkshire wounds over time are shown in Figs. 3, 4, 5, 6. A typical thick Duroc scar following a deep partial-thickness wound of 0.045″ is shown in Fig. 7.

### Animal Tissues

The Institutional Animal Care and Use Committee of the University of Washington approved this study numbered 2322-04. We obtained (Q-Bar Farm, Dayton, OR) 3 female Duroc and 3 female Yorkshire pigs, 6 weeks old, approximately 15 kg. At seven weeks, ten ∼5×5 cm tangential, partial-thickness wounds were created on the back of each anesthetized pig with a Padgett® dermatome (Integra LifeSciences Corporation, Plainsboro, NJ) set to 0.020″, five shallow and five deep partial-thickness. One pass of the dermatome is sufficient for the shallow wounds but two or three passes are necessary to create the deep partial-thickness wounds. Full-thickness wounds were NOT included. The deep and shallow partial-thickness wounds were alternated on the animals to avoid repeatedly placing one wound depth in the same anatomic location. The wounds were allowed to heal without application of topical agents or dressings. At 1, 2, 3, 12 and 20 weeks post-wounding, 1×4 cm surgical biopsies were collected from one shallow and one deep wound on each pig. The biopsies were obtained near the center of the wounds and each wound was biopsied only once. Furthermore, we have now followed two Durocs and one Yorkshire for forty-six weeks.

### Human Tissues

Over the past years we have collected 82 human hypertrophic scar tissue samples. The University of Washington Human Subjects Division approved this study numbered 95-1109-E07 and specifically waived the need for consent since these samples were obtained during surgical reconstruction and would otherwise have been discarded. Most of these were obtained years after injury as surgical reconstruction is usually performed after considerable time has passed. However, three of these human scar samples were obtained 6.7 to 10.8 months after injury and, therefore, are “early” hypertrophic scar as defined by Santucci [51]. These samples were all raised, red and hard. Sample demographics were Black 2, White 1; upper extremity 2, neck 1; times since injury 6.7 months and 10.8 months; and patient ages were 19 and 54. We used these to spot confirm the deep Duroc expression obtained at Week 20 post wounding (as described below).

### Laser Capture Microdissection

The cones and fat domes were described previously and reviewed in the Introduction. Tissue from the deep dermal cones/fat domes was obtained by laser capture microdissection with the Arcturus® AutoPix Laser Capture Microdissection System (Molecular Devices Corporation, Sunnyvale, CA) as previously reported.

### RNA Isolation and Amplification

RNA was extracted with the PicoPure™ Kit (Molecular Devices Corporation, Sunnyvale, CA) and quality monitored with the Agilent 2100 Bioanalyzer (Agilent Technologies, Inc., Santa Clara, CA), the Agilent RNA 6000 Pico chip, and Eukaryote Total RNA Pico. The sample RNA and 500 pg control RNA were amplified with two rounds using the RiboAmpTM HS Kit (Molecular Devices Corporation, Sunnyvale, CA). The complementary RNA was transcribed and labeled with biotinylated UTP and CTP with the Affymetrix IVT Labeling Kit (Affymetrix, Santa Clara, CA, USA). The quality and quantity of labeled complementary RNA was evaluated spectrophotometrically and with the Agilent 2100 Bioanalyzer (Agilent Technologies, Inc., Santa Clara, CA).

### Hybridization of the Affymetrix GeneChips®

The porcine tissue RNA was analyzed using the Porcine GeneChip® and the human tissue RNA using the Human GeneChip® Human Genome U133 plus 2.0. Scanning was performed with GeneChip® Scanner 3000 (Affymetrix, Santa Clara, CA, USA). The quality of the hybridization and overall chip performance was evaluated by visual inspection of the raw scanned data and the quality control measures in the Affymetrix *.RPT report file. Sixty porcine chips were processed (2 breeds * 3 pigs per breed * 2 wound depths * 5 time points) (GSE26095) and three human chips (GSE26213).

### Annotation of the Porcine GeneChip® and Identification of the Human Orthologs

Affymetrix updates the annotation of the Porcine Genechip® quarterly, but does not use all available porcine data and is therefore incomplete. To improve this annotation, we used new tools developed at Iowa State University. The heart of these tools is the ANEXdb database containing an alignment (called the Iowa Porcine Assembly) of ∼1.6 million publically available porcine sequences [52]. This alignment created 140,087 consensus sequences and 103,888 singletons, annotated by BLAST analysis to human and other species. We mapped all new annotations back to the GeneChip® sequences through BLAST analysis. These analyses have provided human orthologs to 16,753 (69%) of the Porcine GeneChip® probesets. Since some probesets match to the same gene, this translates to 10,732 genes.

### Corroboration of the Microarray Data

**Figure 8:**
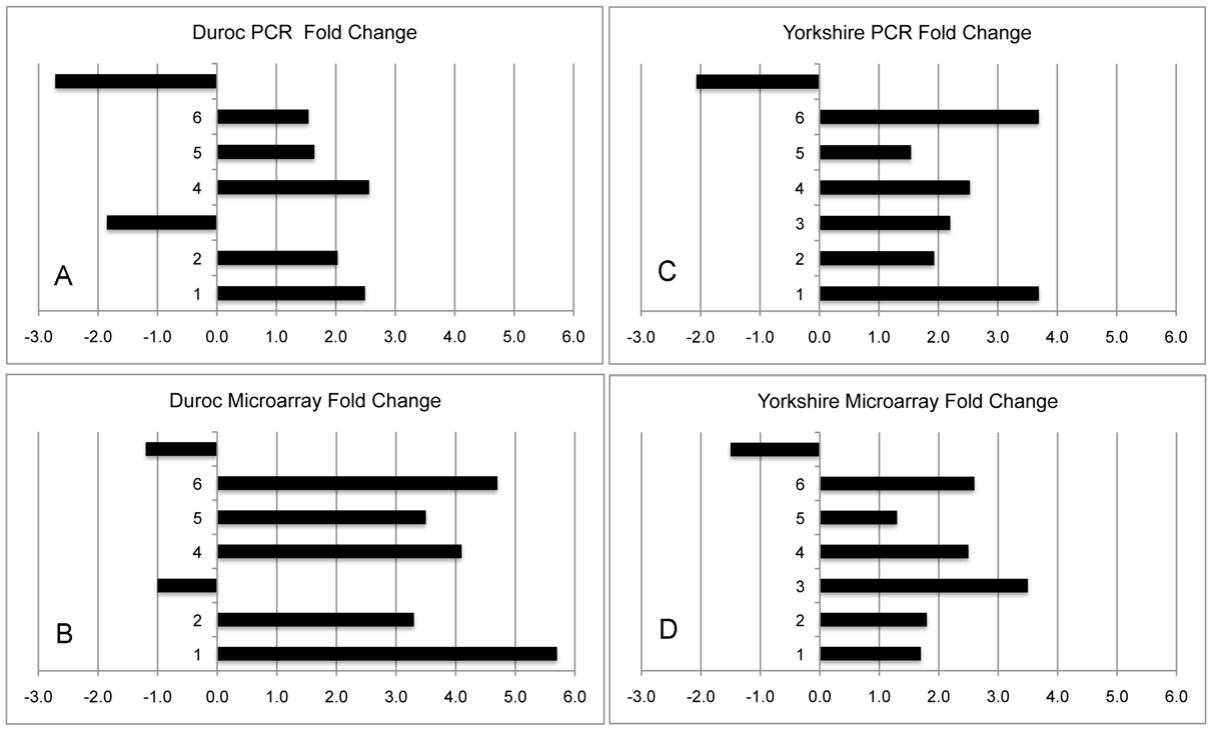
Comparison of PCR and microarray fold changes. Microarray fold changes were verified with qRT-PCR. The PCR fold changes for the genes listed in Table 1 in the Duroc breed (Panel A) are in substantial agreement with the microarray fold changes (Panel B). Similarly, the PCR fold changes in the Yorkshire breed (Panel C) demonstrate substantial agreement with the microarray fold changes (Panel D).

Technical validation of microarray measurements of gene expression was done with qRT-PCR and has been reported [34]. However, we have now performed further qRT-PCR. We selected ten genes at random from Week 3, for which the microarray data of the three bioreplicates for Duroc and Yorkshire were consistent. qRT-PCR was performed as previously described with four technical replicates [34]. In each set of ten, three genes were removed as the PCR-determined fold change was <1.5, the lower limit of detection with the ABI Prism 7900H sequence detection system (Applied Biosystems, Scottsdale, AZ). The genes studied are shown in Table 1 and the results in Fig. 8.

**Table 1:** Genes studied with qRT-PCR.

	Duroc	Yorkshire
1	ANGPTL2	ANGPTL2
2	CDH11	CASP3
3	CSNK2B	CDH11
4	CTSK	CTSK
5	GNAI2	GNAI2
6	IFI30	IFI30
7	OAZ2	MRPS11

A second way to corroborate array data is to examine the control probesets. The GeneChip® includes 188 control probesets. We examined the expression of these controls in the transcriptomes to identify false positives. None of the control probesets indicated differential expression with mixed effects linear regression, indicating that the probability of true negatives becoming false positives is small.

### Data Reduction by Filtering

In accord with recommendations of Bourgon[53] and others we applied nonspecific filters prior to statistical analysis.

1. Control Probesets: The control probesets were removed from further analysis.
2. Affymetrix Present Absent Calls: Those probesets called Absent on all chips were removed from further analysis.
3. Human Ortholog: If the probe set could not be annotated to a human ortholog, it was eliminated from further analysis.
4. Copy Number Dependent Artifacts [54], [55]: It is known that RiboAmp HS yields linear amplification. Nevertheless it is possible that some transcripts respond differently to amplification depending upon copy number. To be sure that all probesets selected for functional genomics evaluation are not affected by copy number, we processed three porcine RNA samples at various dilutions and hybridized the GeneChip® with and without amplification. This clarified which probesets are not affected by copy number; the others were excluded from iterative further analysis. Those selected were either Present in all three dilutions (PPP) or Absent in all three (AAA).
5. Human Expression: The porcine and human genomes and the derivative biology are clearly not identical. Therefore, gene expression in the porcine model must be filtered to match expression in human fibroproliferative scar to be relevant. We used our human scar tissues to confirm the Duroc expression. Since signals between species and between chips cannot be compared, we used the Affymetrix Present Absent calls to accomplish this after discussion with Affymetrix Technical Support. The porcine sample comparable to early human hypertrophic scar, as defined by Santucci[51], is the deep Duroc wound at Week 20. We retained those probes that were PPP or AAA in the human tissue and in the deep Duroc, Week 20 wounds. (Probesets that were A on all 60 chips were removed with filter #2.)
6. Duplicate Genes: For multiple probesets mapping to the same gene, we retained the probeset with the maximum median expression value.

The Porcine GeneChip® contains 24,125 probesets. Table 2 shows the numbers of probesets remaining after each filter was sequentially applied.

**Table 2:** Number of probesets remaining after filters applied sequentially.

Filter	Probesets Remaining After Filter
Control probesets	23,935
All “Absent”	19,109
Human ortholog	13,728
Copy number	9,045
Human match	4,083
Duplicate filter	3,409

### Differential Transcriptomes

There are three independent variables in this system including time, breed (pigment), and wound depth. In addition, the system includes paired measures (shallow and deep partial-thickness wounds on the same animals), and repeated measures (using the same animals at each time point). To control for wound depth, we calculated the log ratio of deep partial-thickness wound expression/shallow partial-thickness wound expression. We then studied breed(pigment) differences of the log ratios over time with mixed linear regression, which accommodates paired measures and repeated measures. This is represented symbolically by ΔtimeΔbreed(pigment) Δdepth. We also studied breed(pigment):time interaction.

We used software R 2.9.2 [56], Bioconductor 2.10.1 [57], [58], affy 1.20.2 [59], gcrma 2.14.1 [60], maanova 1.14.0 [61], and q-value 1.18.0 [62]. We normalized the sixty chips with the affy [59] package and the gcrma [60] algorithm and performed the mixed effects linear regression utilizing the maanova package [61], Fs testing, and 1000 permutations to identify differential expression. We used q-values [62] to limit false discoveries with q≤0.2 considered to be significant.

In addition, if the q-value for regression on breed(pigment) was ≤0.2, we used the maanova package to determine the p-values for the null hypothesis of no breed(pigment):depth differences at each time point and considered ≤0.1 to be significant. This identified the time point(s) at which the differential expression on factor breed(pigment) occurred.

### Functional Genomics Unique to Week 20

It is unknown when the process of fibroproliferative healing begins, clinically or in animal models. It is clear however, in both humans and the Duroc breed that the process is underway at 20 weeks post injury. We applied three functional genomics methodologies to the differential transcriptomes from 1, 2, 3, 12 and 20 weeks and then selected and report those functions, diseases, canonical pathways, ontology terms and gene sets that are unique to Week 20. The three functional genomic approaches included:

1. Ingenuity® Functions, Diseases and Canonical Pathways Analysis: Functional analysis was done with Ingenuity® Pathways Analysis (Ingenuity® Systems, www.ingenuity.com, Application version 8.6, Content version 3003). Parameters were set to default including all data sources and without species filter (analysis finalized on 8/1/2010). Functions and diseases are reported if the p-value<0.05 and the group contained five or more genes. Canonical pathways are reported if the p-value<0.05.
2. Gene Ontology: GoMiner (discover.nci.nih.gov/gominer) [63] was used to identify enriched or over-represented biological processes, cellular components and molecular functions. The parameters included 1) all data sources, 2) human, mouse and rat organisms, 3) evidence codes including TAS, IDA, IMP, IGI, IPI, ISS, and RCA, 4) p and FDR = 0.1, and 5) 1000 randomizations (analysis finalized on 8/1/2010). GO terms are reported if q≤0.20. We also used GOstats version 2.14.0, a package in the R/Bioconductor system [64] with the p-value cutoff set to 0.01. Results of GO Terms with Total Genes ≥ 5 are reported. To ensure robust findings, we report terms found by both applications.
3. Gene Set Analysis: We utilized Gene Set Enrichment Analysis (GSEA(Broad)) [65], [66], [67] and database c2.all.v2.5.symbols.gmt. Parameters were set to default except for min = 5 and permutation type set to “gene set”. Sets were selected if FDR the q-value≤0.20 (analysis finalized on 8/1/2010). We also utilized GSA version 1.03, part of the R/Bioconductor system (http://www-stat.stanford.edu/~tibs/GSA) [68]. Parameters were set to default except for min = 5 and the same gene sets were utilized. We report sets identified by both applications.

## Results

### Differential Transcriptomes

#### 1) Regression on Factor Breed(Pigment)

Mixed effects linear regression on the factor breed(pigment) was accomplished. Selection of those genes for which q<0.2 and for which p<0.1 at one or more time points, returned a differential transcriptome of 162 genes. This differential transcriptome is the core result of this project and includes genes for which the difference between shallow and deep partial-thickness wounds is different between breeds over time. The genes are listed alphabetically in Table S1. The number of genes differentially expressed at each time point is shown in Table 3.

**Table 3:** Number of probesets differentially expressed at each time point.

Week	Number of genes differentially expressed	Over expressed in Duroc tissue	Under expressed in Duroc tissue	1-pi0
1	24	8	16	0.00
2	62	30	32	0.09
3	64	32	32	0.00
12	38	12	26	0.08
20	111	80	31	0.32

#### 2)Regression on Breed(Pigment): Time Interaction

None of the genes that were significant when regressed on breed(pigment) revealed a breed:time interaction, indicating that there is no evidence that the difference between breeds varies at the different time points.

### Functional Genomics Unique to Week 20

We found the functions, diseases, canonical pathways, gene ontology terms and gene sets unique to Week 20. Since collagen must be involved in the process at some point, we noted the presence of collagen genes with differential expression unique to Week 20 in the tables described below.

The Ingenuity® functions unique to Week 20 are summarized in Table 4 and the Ingenuity® diseases unique to Week 20 in Table 5; the complete unabridged data is provided in Table S2. The Ingenuity® canonical pathways unique to Week 20 are shown in Table 6. The GO terms unique to Week 20 found by both GoMiner and GO stats are shown in Table 7.The gene sets unique to Week 20 found by both GSA and GSEA are summarized in Table 8. The entire data set is included in Table S3.

**Table 4:** Ingenuity® functions unique to Week 20 selected if p<0.05 and the function included five or more differentially expressed genes.

Ingenuity® Function	Function Annotation	Collagen Genes with Differential Expression Unique to Week 20
Cardiovascular System Development and Function	Angiogenesis, cardiovascular process of blood vessel, proliferation of endothelial cells	4A1
Cell Morphology	Morphology of cells, morphology of eukaryotic cells, morphology of normal cells	15A1
Cell-To-Cell Signaling and Interaction	Binding of cells, binding of tumor cell lines, signaling of cells	
Cellular Development	Developmental process of tumor cells, growth of breast cancer cell lines	
Cellular Growth and Proliferation	Growth of breast cancer cell lines, proliferation of endothelial cells	4A1
Cellular Movement	Migration of cell lines, migration of endothelial cells, migration of normal cells, migration of tumor cell lines	4A1
Hematological System Development and Function	Coagulation of blood, coagulation of bodily fluid	
Organ Development and Morphology	Morphogenesis of organ	
Organismal Development	Angiogenesis	4A1
Organismal Functions	Coagulation of blood, coagulation of bodily fluid	
Protein Synthesis and Trafficking and Molecular Transport	Localization of protein	
Skeletal and Muscular System Development and Function	Development of muscle	5A3
Tissue Development	Development of muscle, development of tissue	5A3

**Table 5:** Ingenuity® diseases unique to Week 20 selected if p<0.05 and the function included five or more differentially expressed genes.

Ingenuity® Disease	Disease Annotation	Collagen Genes with Differential Expression Unique to Week 20
Cancer	Breast cancer, carcinoma in situ, colorectal cancer, ductal carcinoma, epithelial ovarian cancer, mammary tumor, metastasis of eukaryotic cells, prostate cancer, prostatic carcinoma, prostatic intraepithelial neoplasia, tumorigenesis of cell lines, tumorigenesis of eukaryotic cells	4A1, 15A1
Cardiovascular Disease	Cardiovascular disorder, coronary artery disease	4A1, 15A1
Connective Tissue Disorders	Arthritis, connective tissue disorder, rheumatoid arthritis	4A1, 15A1
Dermatological Diseases and Conditions	Dermatological disorder	4A1, 15A1
Genetic Disorder	Alzheimer's disease, coronary artery disease, epithelial ovarian cancer, prostate cancer, prostatic carcinoma, prostatic intraepithelial neoplasia	4A1, 15A1
Immunological Disease	Autoimmune disease, immunological disorder, rheumatoid arthritis	4A1
Inflammatory Disease	Arthritis, rheumatoid arthritis	4A1
Inflammatory Response	Inflammation	
Neurological Disease	Alzheimer's disease	4A1
Ophthalmic Disease	Ophthalmic disorder	
Psychological Disorders	Psychological disorder	
Skeletal and Muscular Disorders	Arthritis, rheumatoid arthritis	4A1

**Table 6:** Ingenuity® canonical pathways unique to Week 20 selected if p<0.05.

Ingenuity® Canonical Pathways	p-value	Molecules
Bile Acid Biosynthesis	0.039	ALDH2, ACAA1
Complement System	0.039	C1R, CFH
G Protein Signaling Mediated by Tubby	0.047	GNG11, MRAS
Human Embryonic Stem Cell Pluripotency	0.005	FZD4, FGFR1, MRAS, PDGFD, FZD7
PDGF Signaling	0.045	MRAS, CAV1, PDGFD
Riboflavin Metabolism	0.006	TYR, ENPP2
Glioblastoma Multiforme Signaling	0.042	FZD4, MRAS, PDGFD, FZD7
Ovarian Cancer Signaling	0.015	GJA1, FZD4, MRAS, FZD7

**Table 7:** The GO terms unique to Week 20 found by both GoMiner and GOstats (GoMiner q<0.2; GOstats p<0.01 and five or more genes in the term).

Biological Processes	Molecules	Collagen Genes with Differential Expression Unique to Week 20	p-value
GO:0001568 blood vessel development	CAV1, CDH5, FGF18, FGFR1, GJA1, GJA4, LAMA4, MKL2, PTPRM, SERPINF1, TEK		.000
GO:0001944 vasculature development	CAV1, CDH5, FGF18, FGFR1, GJA1, GJA4, LAMA4, MKL2, PTPRM, SERPINF1, TEK		.000
GO:0048514 blood vessel morphogenesis	CAV1, FGF18, FGFR1, GJA1, MKL2, PTPRM, SERPINF1, TEK		.007
**Molecular Functions**			
GO:0001871 pattern binding	CCDC80, CFH, COL5A3, ECM2, LAYN	5A3	.000
GO:0004857 enzyme inhibitor activity	ANXA5, OAZ2, PPP1R2, PROS1, SERPINF1, TWIST1		.001
GO:0005539 glycosaminoglycan binding	CCDC80, CFH, COL5A3, ECM2, LAYN	5A3	.001
GO:0030247 polysaccharide binding	CCDC80, CFH, COL5A3, ECM2, LAYN	5A3	.002

**Table 8:** The gene sets unique to Week 20 found by both GSA and GSEA selected if q<0.2 and five or more genes in the set.

Gene Sets	Brief Set Explanation	Collagen Genes with Differential Expression Unique to Week 20
ADIP HUMAN DN	Down-regulated in primary human adipocytes, versus preadipocytes	
ADIPOGENESIS HMSC CLASS2 UP	Up-regulated 1-14 days following the differentiation of human bone marrow mesenchymal stem cells (hMSC) into adipocytes, versus untreated hMSC cells (Class II)	
APOPTOSIS	Genes involved in apoptosis	
HSA04510 FOCAL ADHESION	Genes involved in focal adhesion	4A1, 5A3
HSA04512 ECM RECEPTOR INTERACTION	Genes involved in ECM-receptor interaction	4A1, 5A3
HSA04664 FC EPSILON RI SIGNALING PATHWAY	Genes involved in Fc epsilon RI signaling pathway	
TGFBETA C1 UP	Upregulated by TGF-beta treatment of skin fibroblasts, cluster 1	
TGFBETA EARLY UP	Upregulated by TGF-beta treatment of skin fibroblasts at 30 min (clusters 1-3)	

### Duroc Scar Thickness At 46 Weeks Post Injury

**Figure 9:**
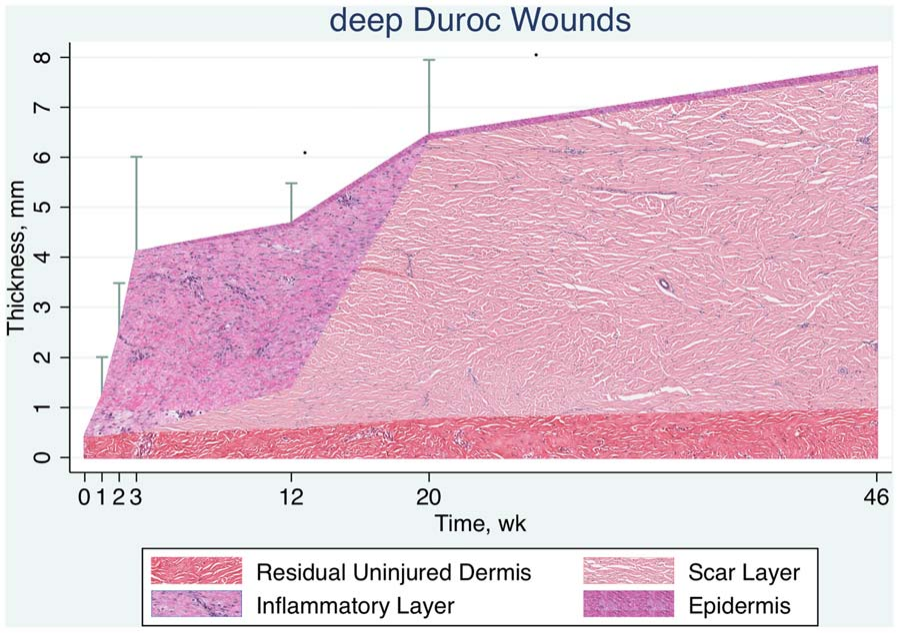
Schematic of time course of wound thickness in deep Duroc wounds. A thick granulation tissue layer is present from wounding to approximately 12 weeks and the scar layer progressively thickens to 46 weeks.

**Figure 10:**
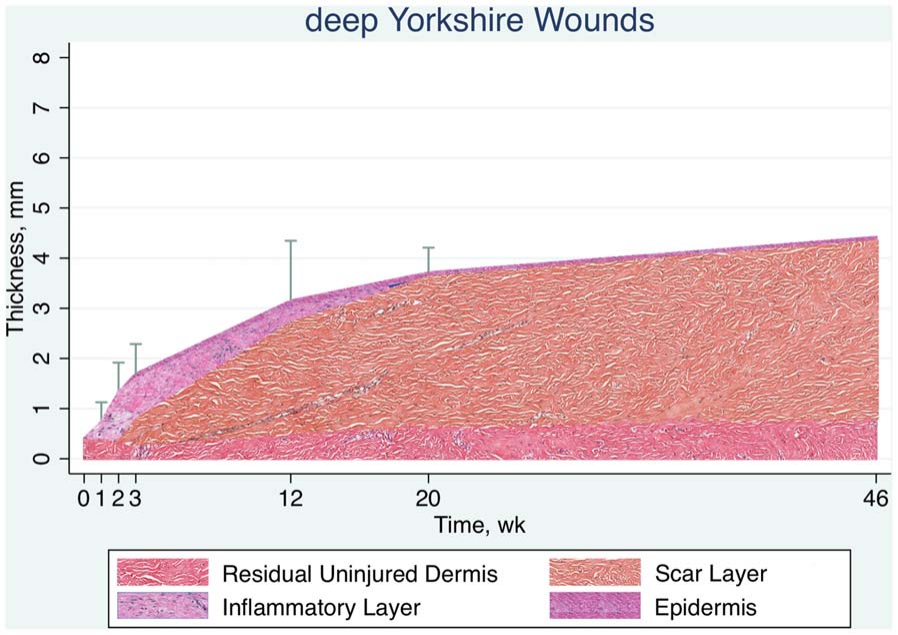
Schematic time course of wound thickness in deep Yorkshire wounds. The granulation tissue layer and the scar layer in the Yorkshire breed are much thinner than in the Duroc breed.

Mean scar thickness in three deep Duroc wounds on each of two Durocs at 46 weeks was 7.8±1.8 mm, compared to 6.3±1.5 mm, previously obtained at Week 20, demonstrating that the scar had not regressed, and was perhaps even increasing in thickness. With this 46-week data and the data from all 14 Durocs and five Yorkshires, schematic mean thickness by time for Duroc and Yorkshire deep wounds is shown in Figs. 9 and 10. The Duroc breed produces considerably more granulation tissue and scar than the Yorkshire breed.

## Discussion

### Differential Transcriptome

The fundamental, core output of this project is the differential transcriptome provided in Table S1. These are the genes that were differentially expressed controlling for wound depth and breed(pigment) over time. It is a reasonable assumption that the transcriptional aspect of the systems biology of fibroproliferative scarring is contained within this collection, to be teased out for interrogation with traditional reductionist methods.

One immediate observation is that the types of collagen implicated differs from our previous report[34], In that study we suggested that collagens I, III, IV, V, VI, VII, XIV and XV were differentially expressed, whereas here we report only collagens IV, V and XV. The filters and statistical criteria in this manuscript are far more stringent than in the previous manuscript and the probesets for types I, III, VI, VII and XIV failed to pass. It is interesting that three of the “lesser” collagens did pass the more stringent criteria and may be the key to the collagen disorder in fibroproliferative scarring.

A second immediate observation relates to genes involved in cutaneous pigment. It is clinically quite clear that the incidence of hypertrophic scarring increases with increasing skin pigmentation. Four genes known to be involved in skin pigmentation were differentially expressed including FZD4, FZD7, SLC24A5 and TYR. Their potential involvement in fibroproliferative healing warrants exploration.

A third observation is that the number of genes differentially expressed at Week 20 and the values of 1-pi0 (see Table 3, pi0 is an estimate of the proportion of non-differentially expressed genes) suggest that there is surge of differential expression at this time point. This has three possible explanations: 1) laboratory error or chance, 2) simple progression and biological magnification of a process that started much earlier, or 3) an event that began and ballooned after Week 12. Given that the pi0 values for Weeks 1-12 are quite low, we favor explanation #3. This, if true, has significant ramifications as it implies that the aberrant process begins quite late. It would then be related to events of late wound remodeling rather than early wound healing and a different set of biological events would be suspect.

### Functional Genomics Unique to Week 20

Several immediate observations may also be made on the functional genomic results unique to Week 20. First, the Ingenuity® functions, GO terms and gene sets found from the expression data confirm and are confirmed by studies done long ago with very different methodologies. Kischer implicated mast cells in 1972 [69] and angiogenesis in 1982 [70]. Massague reviewed the TGFß family in 1990 [71]. Wassermann reported differential expression of apoptotic genes in 1998 [72]. Dabiri [73] recently implicated focal adhesions. This agreement further confirms the validity of the model.

Other observations/questions include: Inflammatory disease (Table 5) and the inflammatory response (Table 5) appear in the findings. Inflammation has long been known to be part of fibrosis [74] but why are the genes involved differentially expressed at Week 20 and not before?

Angiogenesis appeared in the findings (Table 4, 7). That angiogenesis is involved is not surprising, and was implicated by Kischer thirty-eight years ago [70]. But, as for inflammation, why are the genes involved differential at Week 20 and not before?

The similarity to various cancers is evident (Table 5). That wound healing shares events with cancer has also been known for some time, but again why does the similarity appear at Week 20? And might the similarity lead to control of fibroproliferative healing?

Adipogenesis appears in the outcomes (Table 8) and adipose tissue derived stem cells are increasingly a part of wound healing studies. Are events in the fat dome driving the fibroproliferation?

And finally, does the similarity to autoimmune rheumatoid arthritis appearing at Week 20 (Table 5) provide a clue to etiology?

These and many other questions will emerge from these early functional genomic results. The transcriptome will now have to be searched for “answers” to be interrogated in the laboratory.

### Duroc Scar Thickness At 46 Weeks Post Injury

One of the challenges to this porcine model has been that the scar may be thick at twenty weeks but has matured away by one year, unlike human hypertrophic scar. The observation that the porcine thick scar has not disappeared at Week 46 and, in fact, may be thicker indicates that, as in humans, the porcine scar is long-lived.

### System Variance

This is a complex system and there are many sources of variance.

1. Purity of the breed of the pigs: The producer verified the animals as representing the breeds; we required that the pigs also match the phenotypic requirements of the National Swine Registry.
2. Variable wound depth and site of biopsy within the wound: Creation of tangential wounds with a dermatome is not precise. However, we have created > 200 such wounds and so have considerable experience. The wounds were biopsied in the center where depth is most uniform.
3. Pigs “snuffling” on wounds: The pigs were separated for 3–5 days post wounding.
4. Infection: With our open wound model, infection occurred in only 1 of 20 pigs, 1 in ∼200 wounds. This wound is not included in this report.
5. RNA degradation: We have addressed this with the following: 1) time from excision to snap freeze, accomplished in <10 minutes, 2) time for cryosection, accomplished in <20 minutes, and 3) time for laser capture microdissection, accomplished in this fibrous tissue in <45 minutes, very analogous to Ryge [75]. In addition, King has demonstrated that the biological variability is greater than the variance introduced here [76].
6. Multiple cell types in the laser microdissected tissues: This is by design and of necessity, since at this point we do not know which cell type(s) are involved in the process.
7. Dermatome wound model: This model, as described by Silverstein [77], [78], uses wounds created with a dermatome, not thermal injury. However, it is well known to clinicians that deep donor sites and deep abrasions frequently develop hypertrophic scars.
8. Genes not on the chip or not annotated: It is possible that the genes involved in fibroproliferative healing are not on the current Affymetrix® Porcine GeneChip®. However, the chip contains approximately two thirds of the porcine genome, so is likely to provide clues to the causes. Another possibility is that the genes involved in cutaneous fibrosis are not annotated. We kept the GeneChip® annotated with updated information.
9. False positives/negatives: We controlled false positives as described and since we searched for expression patterns made up of several genes, not individual genes, false negatives are unlikely.

### Summary

Wynn has nicely reviewed and summarized the current understanding of tissue fibrosis[74] from whom we quote:

*Most chronic fibrotic disorders have in common a persistent irritant that sustains the production of growth factors, proteolytic enzymes, angiogenic factors and fibrogenic cytokines, which stimulate the deposition of connective tissue elements that progressively remodel and destroy normal tissue architecture.*

The clues we here report may identify the “persistent irritant”. The differential transcriptome, refined by time span, wound depth and pigment; the appearance of the “lesser” collagen genes (4, 5 and 15); the overlap of transcriptome and pigment genes; and the related functions, diseases, canonical pathways, GO terms and gene sets together may lead to a differential transcriptome-driven hypothesis to explain and to treat the fibroproliferative condition.

## Supporting Information

Table S1 Gene Sets Uniquely Significant at Week 20. Columns 4 and 5 are the regression p and q values. Columns 6–10 are the p-values for each time point and whether the gene was differentially over or under expressed in Duroc compared to Yorkshire. Empty cells indicate that the weekly p-values did not achieve significance.
(XLS)

Table S2 Functions and Diseases Uniquely Significant at Week 20.
(XLS)

Table S3 Gene Sets Uniquely Significant at Week 20.
(XLS)
