# Correction: Functional Genomics Unique to Week 20 Post Wounding in the Deep Cone/Fat Dome of the Duroc/Yorkshire Porcine Model of Fibroproliferative Scarring

**DOI:** 10.1371/journal.pone.0123368

**Published:** 2015-04-02

**Authors:** The PLOS ONE Staff

The title for S1 Table is incorrect. It should read: Differential transcription on factor breed. Please view the correct S1 Table below.

## Supporting Information

S1 Table Gene Sets Uniquely Significant at Week 20. Columns 4 and 5 are the regression p and q values. Columns 6–10 are the p-values for each time point and whether the gene was differentially over or under expressed in Duroc compared to Yorkshire. Empty cells indicate that the weekly p-values did not achieve significance.
(XLS)

## References

[1] Engrav LH, Tuggle CK, Kerr KF, Zhu KQ, Numhom S, Couture O, et al (2011) Functional Genomics Unique to Week 20 Post Wounding in the Deep Cone/Fat Dome of the Duroc/Yorkshire Porcine Model of Fibroproliferative Scarring. PLoS ONE 6(4): e19024 doi:10.1371/journal.pone.0019024 21533106 10.1371/journal.pone.0019024 PMC3080398

